# MtDNA‐depleted neuronal cell transcriptomes reveal Alzheimer's disease‐related changes

**DOI:** 10.1002/alz.70929

**Published:** 2025-11-26

**Authors:** Blaise W. Menta, Emily Schueddig, Amol Ranjan, Yanming Li, Shea J. Andrews, Heather M. Wilkins, Dong Pei, Russell H. Swerdlow

**Affiliations:** ^1^ University of Kansas Alzheimer's Disease Research Center Kansas City Kansas USA; ^2^ Department of Biochemistry & Molecular Biology University of Kansas Medical Center Kansas City Kansas USA; ^3^ Department of Biostatistics & Data Science University of Kansas Medical Center Kansas City Kansas USA; ^4^ Department of Psychiatry & Behavioral Sciences University of California San Francisco San Francisco California USA; ^5^ Department of Neurology University of Kansas Medical Center Kansas City Kansas USA; ^6^ Department of Cell Biology & Physiology University of Kansas Medical Center Kansas City Kansas USA; ^7^ Department of Pharmaceutical Chemistry University of Kansas Lawrence Kansas USA

**Keywords:** Alzheimer's disease, mitochondria, mitochondrial DNA, RNA sequencing, transcriptome

## Abstract

**INTRODUCTION:**

We determined whether mitochondrial DNA (mtDNA) depletion induced Alzheimer's disease (AD)‐relevant transcription changes.

**METHODS:**

Following RNA sequencing (RNA‐seq), we identified differentially expressed genes (DEGs) between SH‐SY5Y or NT2 mtDNA‐depleted (ρ0) and intact (ρ+) cell lines and quantified concordant DEG changes. Gene set enrichment analysis and over‐representation analysis were used to determine the impact on the Kyoto Encyclopedia of Genes and Genomes (KEGG) AD and other neurodegenerative disease pathways, ascertain pathway and term enrichment in the Reactome and Gene Ontology databases, and generate Ingenuity Pathway Analysis *z*‐scores.

**RESULTS:**

Relative to their ρ+ comparators, ρ0 lines differentially expressed >75% of their genes. The KEGG AD pathway was significantly enriched, and equivalently altered genes ranked the AD, Parkinson's disease, ALS, and Huntington's disease KEGG pathways among the most enriched gene sets. AD‐related enriched pathways and terms reflected lipid, insulin signaling, synapse, inflammation/immune response, endosome/endocytosis, RNA, and proteostasis biology.

**CONCLUSION:**

MtDNA depletion alters gene expression in ways that recapitulate or predictably promote AD molecular phenomena.

**Highlights:**

- MtDNA‐depleted neuronal cell lines reshuffle nuclear gene expression.
- The KEGG AD pathway is enriched with DEGs.
- Transcription‐defined pathways and terms relating to AD biology broadly change.

## 1 BACKGROUND

Mitochondrial dysfunction is a recognized feature of Alzheimer's disease (AD).^1^ It is observed in the brain and multiple non‐brain tissues of AD subjects.^2^ Because mitochondrial efficiency declines with age and to a greater extent in AD, and advancing age represents AD's most profound risk factor, some suspect mitochondrial function could link brain aging to AD.^1^, ^3^ In experimental models, primary mitochondrial dysfunction alters amyloid precursor protein (APP), amyloid beta (Aβ), microtubule‐associated protein tau (MAPT), and apolipoprotein E (APOE) biology,^4^, ^5^, ^6^, ^7^ which suggests mitochondrial dysfunction could perhaps even initiate the disease, although data from other models show APP, Aβ, MAPT, and APOE can also initiate mitochondrial dysfunction.^8^

Omics studies further position mitochondria within the AD biology hierarchy. Genomic, proteomic, and transcriptomic studies independently suggest a critical role.^9^, ^10^, ^11^, ^12^, ^13^ Beyond demonstrating changes to mitochondrial genes, proteins, and pathways, omics studies reveal other brain‐localized molecular changes, including widespread perturbations in synapse, immune response, lipid metabolism, structural stabilization, and proteostasis function.^11^ Patient autopsy tissue studies, though, can emphasize non‐specific changes that reflect a state of end‐stage pathology or the status of atypical, cell‐type‐specific, or exceptional cells that resist the disease or otherwise persist for unclear reasons. Stand‐alone omics studies are also descriptive and hypothesis‐generating, as opposed to directly mechanistic and hypothesis testing.

To leverage and enhance the utility of an omics approach, we performed a transcriptomic analysis of human neuronal mitochondrial DNA (mtDNA)‐depleted (ρ0) cell lines. Our intent was to use these lines to model a specific and chronic form of primary mitochondrial dysfunction and determine if it initiated transcriptional changes seen in AD subject tissues or initiated transcriptional changes that relate to known AD phenomena. When considering the scope and nature of the transcriptional changes we report, we emphasize our intent was to use ρ0 cell lines not as models of AD, but rather as models of primary mitochondrial dysfunction.

## 2 METHODS

### 2.1 Cell lines and cell culture

SH‐SY5Y and NTERA‐2/NT2 (NT2) mtDNA‐depleted (ρ0) cell lines were generated by exposing the parent (ρ+) lines to ethidium bromide for prolonged durations before transferring them to media that did not contain ethidium bromide.^14^, ^15^ For this study, SH‐SY5Y ρ0 and ρ+ cells were maintained in DMEM containing 25 mM glucose (Gibco Catalog No.: 11965) supplemented with 10% fetal bovine serum (FBS), 1% penicillin/streptomycin, 100 µg/mL sodium pyruvate, and 50 µg/mL uridine. The NT2 ρ0 and ρ+ cells were maintained in DMEM containing 25 mM glucose (Gibco Catalog No.: 11965) supplemented with 10% FBS, 1% penicillin/streptomycin, 200 µg/mL sodium pyruvate, and 150 µg/mL uridine. Cells were kept in a 5% CO_2_ humidified incubator at 37°C, with media exchanges performed every other day for cultures below 50% confluence and daily for those above 50% confluence.

**RESEARCH IN CONTEXT**

1. **Systematic review**: We reviewed literature detailing omics‐level gene expression, pathway, and term changes in AD. This literature revealed changes to molecular pathways and terms that reflect lipid, insulin signaling, inflammation/immune response, synapse, endosome/endocytosis, RNA, and proteostasis biology.
2. **Interpretation**: Analyses of RNA‐seq data from mtDNA‐depleted (ρ0) and mtDNA‐intact (ρ+) cell lines found that relative to their ρ+ comparators, ρ0 lines differentially expressed >75% of their genes. These DEGs were enriched in the KEGG AD pathway and other neurodegenerative disease pathways. AD‐related enriched pathways and terms reflected lipid, insulin signaling, inflammation/immune response, synapse, endosome/endocytosis, RNA, and proteostasis biology.
3. **Future directions**: The impact of other types of mitochondrial dysfunction on AD molecular phenomena warrants investigation. The extent to which mitochondrial dysfunction initiates molecular changes that recapitulate or promote AD molecular phenomena can inform the viability of a mitochondrial cascade hypothesis that proposes mitochondrial dysfunction promotes AD.

### 2.2 RNA library generation, RNA library validation, and mRNA‐seq

RNA isolations from our ρ0 and ρ+ cell lines were performed using a Qiagen miRNeasy Mini kit (217004) with on‐column DNase treatment using a Qiagen RNase‐Free DNase Set (Catalog No. 79254). The stranded RNA‐seq was performed using an Illumina NovaSeq 6000 Sequencing System at the University of Kansas Medical Center Genomics Core (Kansas City, KS, USA). Quality control of the total RNA submissions was completed using an Agilent TapeStation 4200 using an RNA ScreenTape Assay kit (Agilent Technologies, Catalog No. 5067‐5576). Total RNA (1 ug) was used to initiate the library preparation protocol. The total RNA fraction was processed by oligo dT bead capture of mRNA, fragmentation of enriched mRNA, reverse transcription into cDNA, end repair of cDNA, ligation with the appropriate Unique Dual Index (UDI) adaptors, and strand selection and library amplification by polymerase chain reaction (PCR) using a Universal Plus mRNA‐seq with NuQuant library preparation kit (Tecan Genomics, Catalog No. 0520‐A01).

Library validation was performed using a D1000 ScreenTape Assay kit (Agilent Technologies, Catalog No. 5067‐5582) on an Agilent TapeStation 4200. Concentration of each library was determined with the NuQuant module of the library prep kit using a Qubit 4 Fluorometer (Thermo Fisher Scientific/Invitrogen), libraries were normalized to 4 nM concentration and pooled. The multiplexed pool was quantitated, in triplicate, using a Roche Lightcycler96 with FastStart Essential DNA Green Master (Roche, Catalog No. 06402712001) and KAPA Library Quant (Illumina) DNA Standards 1‐6 (KAPA Biosystems, Catalog No. KK4903). Using the quantitative PCR results, the RNA sequencing (RNA‐seq) library pool was adjusted to 1.9 nM for multiplexed sequencing.

Pooled libraries were denatured with 0.2 N NaOH (0.04 N final concentration) and neutralized with 400 mM Tris‐HCl pH 8.0. A dilution of the pooled libraries to 380 pM was performed in the sample tube, on the instrument, followed by onboard clonal clustering of the patterned flow cell using a NovaSeq 6000 S1 Reagent Kit version 1.5 (200 cycle) (Illumina, Catalog No. 20028318). A 2 × 101 cycle sequencing profile with dual index reads was completed using the following sequence profile: Read 1–101 cycles × Index Read 1–8 cycles × Index Read 2–8 cycles × Read 2–101 cycles. Following collection, sequence data were converted from .bcl file format to fastq file format using bcl2fastq software and de‐multiplexed into individual sequences for data distribution using a secure file transfer protocol site for further downstream analysis.

### 2.3 RNA‐seq data preprocessing and quality control

The quality of the raw FastQ files was first assessed using the FastQC pipeline. Reads were mapped, and transcript abundance was quantified at the gene level using RNA‐seq by Expectation Maximization (RSEM) with the bowtie2 aligner and the human genome assembly hg38. Raw gene counts were then normalized according to library size. After filtering lowly expressed genes, 15,914 and 16,029 genes were retained for downstream statistical analysis in the SH‐SY5Y and NT2 cell lines, respectively. There were 10 ρ0 and 10 ρ+ samples per cell line, bringing the total sample size to 40. The RNA‐seq data in this publication have been deposited in NCBI's Gene Expression Omnibus (GEO) and are accessible through GEO Series accession number GSE309664.

### 2.4 Differential expression analysis

The R package edgeR (https://bioconductor.org/packages/release/bioc/html/edgeR.html) was used for the gene differential expression (DE) analysis. EdgeR implements a range of statistical methodologies based on negative binomial distributions, including empirical Bayes estimation, exact tests, generalized linear models, and quasi‐likelihood tests. EdgeR DE analysis was performed independently on the SH‐SY5Y and NT2 cell lines, in which we compared each ρ0 line to its parental ρ+ line. The magnitude of difference between groups was quantified as the log_2_‐transformed fold change (FC). Multiple testing adjustment was performed by correcting the raw *p* values using the Benjamini–Hochberg procedure to control the false discovery rate (FDR). We refer to our corrected *p* values as the FDR, with FDR ≤ 0.05 considered statistically significant.

### 2.5 Equivalent Change Index

To measure genes that are changing to the same degree in the SH‐SY5Y and NT2 cell lines, we used the Equivalent Change Index (ECI).^16^ The ECI is a statistic that quantifies the degree of equivalent or inverse change for a single gene across two experiments. An ECI of 1 means the effect size was exactly the same for a gene in both cell lines, whereas an ECI of −1 means the effect size was exactly opposite in both cell lines. We used the log_2_ FC from comparing gene expression in ρ0 to ρ+ cells as the effect size for computing the ECI.

### 2.6 Kyoto Encyclopedia of Genes and Genomes (KEGG) database analyses

The KEGG pathway enrichment analysis was performed using the R package fgsea, with gene sets obtained from the MSigDB (Molecular Signatures Database) version 7.1 collection c2.cp.kegg.v7.1.symbols.gmt (available at: https://data.broadinstitute.org/gsea‐msigdb/msigdb/release/7.1/). This gene set file comprises curated KEGG pathways and is widely used for gene set enrichment analysis (GSEA). We utilized a pre‐ranked list of edgeR‐derived *t* statistics comparing ρ0 and ρ+ cells as the input gene list. Enrichment was assessed against KEGG pathways with gene set sizes ranging from 15 to 500.

To test for pathway enrichment of differentially expressed genes (DEGs) with highly equivalent changes, GeneCodis 4 was used to perform an overrepresentation analysis (ORA) within the KEGG database. The 5000 DEGs with the highest ECI values served as the input set of genes.

### 2.7 GSEA and ORA using Reactome and Gene Ontology databases

To avoid applying a magnitude‐of‐change cutoff to the significant genes, we used GSEA. We specifically did this to capture the small, but coordinated, changes in expression that would be typically missed when having to choose a threshold. GSEA was performed using the clusterProfiler R package using the Reactome pathway database and Gene Ontology (GO) resource. GO terms for Biological Process, Cellular Component, and Molecular Function were tested independently. The input gene list was ranked using log_2_ FC values from comparing expression in ρ0 to ρ+ cells. Next, to focus on pathways perturbed by genes with large changes in expression, an ORA was performed using clusterProfiler with the Reactome pathway database and GO terms. DEGs with FDR ≤ 0.05 and absolute log_2_ FC ≥ 1 were considered as the input set, and the background genes were set to only the genes tested for DE (15,914 and 16,029 genes, respectively, for SH‐SY5Y and NT2 cells). Multiple testing adjustment was performed by correcting the raw *p* values using the Benjamini–Hochberg procedure to control the FDR. Pathways and terms with FDR ≤ 0.05 were considered significantly enriched pathways.

Due to the hierarchical nature of the GO resource, many significant terms can be semantically similar, which leads to redundancy in results. To cut down on redundant terms, we used the simplify function in clusterProfiler with the parameters measure = “Wang” and cutoff = 0.8. To distinguish database‐specific, named pathways and terms from general references to biological phenomena, named pathways and terms are italicized when stated in the text.

### 2.8 Ingenuity pathway analyses

We performed a QIAGEN Ingenuity Pathway Analysis (IPA) that used ORA to determine significantly enriched pathways. DEGs with FDR ≤ 0.05 and absolute log_2_ FC ≥ 1 were considered as the input set, and the background genes were set to only the genes tested for DE (15,914 and 16,029 genes, respectively, for the SH‐SY5Y and NT2 cell lines). Multiple testing adjustment was performed by correcting the raw *p* values using the Benjamini–Hochberg procedure to control the FDR. Pathways with FDR ≤ 0.05 were considered significantly enriched pathways.

IPA also determines *z*‐scores to predict pathway activation status. A *z*‐score ≥ 2 was considered statistically significant activation, and a *z*‐score ≤ −2 statistically significant inhibition in ρ0 compared to ρ+ cells. Named pathways are italicized when stated in the text.

## 3 RESULTS

### 3.1 mtDNA depletion profoundly alters gene expression

To verify the mtDNA‐depletion status of our SH‐SY5Y and NT2 ρ0 cells, we considered the state of expression of the 13 mtDNA‐localized respiratory chain genes. We saw profoundly reduced expression for each of those genes to an extent that is consistent with an absence of expression and that confirms the integrity of our models (Table S1).

**FIGURE 1:**
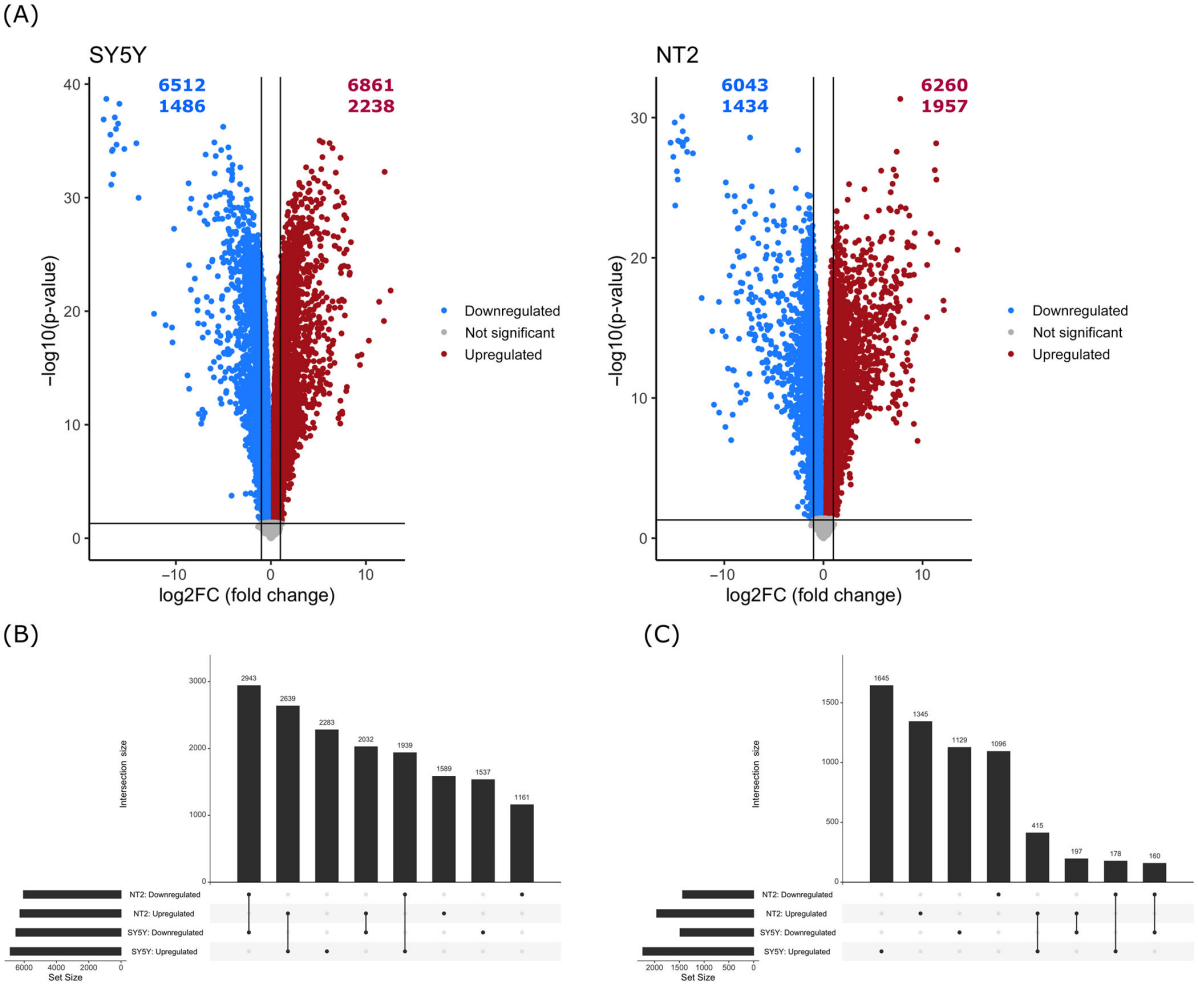
Volcano plots and summary of concordant/divergent gene expression. (A) Volcano plots from the SH‐SY5Y ρ0 versus ρ+ and NT2 ρ0 versus ρ+ RNA sequencing. The upper number in each panel shows the total number of all differentially expressed genes (DEGs) with a false discovery rate (FDR) < 0.05. The lower number in each panel shows the total number of all DEGs with FDR < 0.05 and absolute log_2_ fold change (FC) ≥ 1. (B) Concordant and divergent DEGs considering all genes with FDR < 0.05. (C) Concordant and divergent DEGs considering all genes with FDR < 0.05 and absolute log_2_ FC ≥ 1.

We detected more DEGs than autopsy brain or other cell‐based studies. We suspect the homogeneity of our in vitro system facilitated a more sensitive screen for DEGs than more biologically heterogeneous and variably collected autopsy brains. The magnitude of our experimental manipulation was also robust, which could elicit more DEG changes than milder manipulations performed in other cell‐based studies. Table 1 summarizes the number and proportion of DEGs. After filtering minimally expressed genes, we identified a total of 15,914 genes expressed in the SH‐SY5Y cell line and 16,029 genes expressed in the NT2 cell line. The SH‐SY5Y ρ0 cells had 13,373 DEGs (84% of the total gene count), with 6861 upregulated and 6512 downregulated genes. The NT2 ρ0 cells had 12,303 DEGs (77% of the total gene count), with 6260 upregulated and 6043 downregulated genes (Figure 1A and Table S2). After considering only those DEGs that showed an absolute log_2_ FC ≥ 1, the SH‐SY5Y ρ0 cells had 3724 DEGs (23% of the total gene count), with 2238 upregulated and 1486 downregulated genes. Applying the more stringent absolute log_2_ FC ≥ 1 cutoff to the NT2 ρ0 cells revealed 3391 DEGs (21% of the total gene count), with 1957 upregulated and 1434 downregulated genes.

**TABLE 1:** Summary of individual gene and pathway expression changes.

Method	DEGs (FDR < 0.05)	DEGs (FDR < 0.05 and absolute log_2_FC ≥ [1]	ORA	GSEA	ORA	GSEA	*Z*‐score (absolute *z*‐score ≥ [2])
DATA/DATABASE	RNASeq (percentage of all genes)	RNASeq (percentage of all genes)	Reactome	Reactome	GO (BP; CC; MF)	GO (BP; CC; MF)	IPA
SH‐SY5Y ρ0 cell‐altered genes or pathways	13,373 (84% of 15,914 genes)	3724 (23% of 15,914 genes)	84	256	694 (BP 554; CC 67; MF 73)	503 (BP 330; CC 96; MF 77)	78
NT2 ρ0 cell‐altered genes or pathways	12,303 (77% of 16,029 genes)	3391 (21% of 16,029 genes)	58	24	379 (BP 289; CC 47; MF 43)	173 (BP 88; CC 55; MF 30)	102
Concordantly altered genes or pathways	5582	575	29	11	206 (BP 144; CC 29; MF 33)	88 (BP 39; CC 32; MF 17)	

Abbreviations: BP, biological process; CC, cellular component; DEG, differentially expressed genes; FDR, false discovery rate; GO, Gene Ontology; GSEA, gene set enrichment analysis; IPA, Ingenuity Pathway Analysis; MF, molecular function; ORA, overrepresentation analysis.

For all DEGs, the SH‐SY5Y ρ0 and NT2 ρ0 cells shared 5582 concordant (upregulated or downregulated in both cell lines) and 3971 divergent (upregulated in one cell line but downregulated in the other) DEGs. For DEGs that exceeded the absolute log_2_ FC ≥ 1 cutoff, the SH‐SY5Y ρ0 and NT2 ρ0 cells shared 575 concordant and 375 divergent DEGs (Figure 1B, C).

To determine whether the SH‐SY5Y and NT2 ρ0 versus ρ+ cell line concordant DEGs have similar or different degrees of change, we used the ECI approach. The calculated ECI for the 5582 concordant genes ranged from 0.0155 to 0.9998, indicating that although genes may be upregulated or downregulated in both cell lines, the degree to which they are changing is not the same. There were 465 genes exhibiting highly equivalent change (ECI ≥ 0.9) in ρ0 compared to ρ+ in both cell lines (Table S3). Table 2 lists the 20 genes with the highest calculated ECI values. The functional roles for some of these genes pertain to RNA homeostasis, lipid biology, protein homeostasis, and endocytosis. One of the genes, PARK7, is alternatively known as DJ‐1, and mutations in this gene cause autosomal recessive Parkinson's disease.^17^

**TABLE 2:** SH‐SY5Y and NT2 genes with the highest ECI scores.

Gene	ECI	SH‐SY5Y Log_2_FC	SH‐SY5Y FDR	NT2 Log_2_FC	NT2 FDR	Function notes
TATDN2	0.999835	−0.4071	0.000000	−0.4072	0.000000	Resolves RNA R‐loops
C1S	0.999583	2.4044	0.000000	2.4054	0.000000	Complement pathway
EIF2AK1	0.999336	−0.4304	0.000000	−0.4301	0.000000	Proteostatic stress/translation
P3H1	0.999038	0.5090	0.000000	0.5095	0.000000	Collagen synthesis
TRMT1L	0.998923	−0.5191	0.000000	−0.5197	0.000000	tRNA modification
MFSD12	0.998849	−0.2539	0.000001	−0.2536	0.000009	Cysteine transport, cell cycling
AC006548.28	0.998459	0.3663	0.000003	0.3658	0.000007	lncRNA
PEF1	0.998233	−0.6891	0.000000	−0.6879	0.000000	ER‐Golgi transport
TMA7	0.998210	−0.4842	0.000000	−0.4851	0.000000	Cell growth; mitochondria
SNX22	0.998045	−0.8716	0.000000	−0.8699	0.000000	Endocytosis; intracellular trafficking
ADPRHL2	0.997978	−0.8716	0.000000	−0.4366	0.000001	DNA damage
CDK7	0.997864	−0.4357	0.000000	−0.4729	0.000000	Transcription regulator
HCCS	0.997864	−1.0399	0.000000	−1.0376	0.000000	Mitochondrial lipid regulation, respiration
SUCLG2‐AS1	0.997681	0.7395	0.000034	0.7411	0.000073	TCA cycle
ELK4	0.996702	0.2654	0.000460	0.2657	0.002252	Transcription factor; Ras‐ERK related
PAPOLA	0.996563	−0.3250	0.000000	−0.3239	0.000000	mRNA stability
IDH3B	0.995377	−0.7849	0.000000	−0.7813	0.000004	TCA cycle
WFS1	0.995145	0.8360	0.000000	0.8401	0.000000	Endoplasmic reticulum (ER) calcium handling; ER stress response
EGFL8	0.995112	0.8410	0.000009	0.8451	0.000000	Cell–cell interaction; MAP‐ERK related; differentiation
PARK7 (DJ‐1)	0.994829	−0.2751	0.000005	−0.2737	0.000013	Oxidative stress; DNA damage

### 3.2 mtDNA depletion alters the expression of AD‐associated genes

We considered the impact of mtDNA depletion‐induced primary mitochondrial dysfunction on the expression of genes that encode or give rise to proteins that aggregate in AD brains, are implicated in AD through linkage, or are tied to AD through genome‐wide association studies (GWASs).^18^, ^19^, ^20^ Table 3 summarizes the expression status of those genes in our SH‐SY5Y and NT2 ρ0 cells. Both cell lines showed frequent minor but significant (absolute log_2_ FC < 1; FDR ≤ 0.05) expression changes that were variably concordant or divergent between the lines. There were occasional robust changes (absolute log_2_ FC ≥ 1; FDR ≤ 0.05) that affected one cell line or another, for example, expression of the lipid biology‐related APOE gene^21^ was sharply increased in the SH‐SY5Y ρ0 cells, while the expression of the endocytosis‐related BIN1 gene^22^ was strongly reduced in the NT2 ρ0 cells. Two genes showed a robust concordant expression change across both lines. One was SNCA, which encodes the α‐synuclein protein that frequently accumulates within neurons in those with AD (SH‐SY5Y ρ0 log_2_FC = 1.467; NT2 ρ0 log_2_FC = 2.729).^23^ The other was CLU, which reportedly influences amyloid processing, lipid metabolism, and synaptic function (SH‐SY5Y ρ0 log_2_ FC = 1.714; NT2 ρ0 log_2_FC = 1.300).^24^

**TABLE 3:** Genes with known or potential relevance to AD based on disease histopathology, linkage studies, or genome‐wide association studies.

Gene	SH‐SY5Y ρ0 Cells		NT2 ρ0 Cells	
	Log_2_FC	FDR	Log_2_FC	FDR
APP	0.956	3.87E‐18	0.150	3.18E‐03
MAPT	0.770	5.26E‐14	−0.493	1.82E‐02
APOE	**5.423**	1.84E‐31	0.264	NS
PSEN1	0.579	2.30E‐13	0.368	6.00E‐07
PSEN2	0.058	NS	0.394	5.49E‐07
SORL1	0.656	2.52E‐09	0.489	1.70E‐04
SNCA	**1.467**	7.00E‐20	**2.729**	8.78E‐17
TARDBP	−0.363	3.19E‐13	−0.280	7.91E‐09
TREM2	ND	ND	ND	ND
CTSB	**1.539**	3.91E‐26	−0.872	3.67E‐10
SHARPIN	0.275	5.92E‐06	−**1.260**	6.62E‐14
ANKH	0.194	8.01E‐07	−0.353	1.03E‐06
SEC61G	−0.782	9.82E‐14	−0.441	7.20E‐07
WDR12	−0.164	1.85E‐05	−0.168	9.73E‐03
ABCA1	ND	ND	−0.934	3.74E‐03
TPCN1	−0.015	NS	−0.096	NS
MME	−**5.300**	1.11E‐17	**2.367**	1.29E‐14
CASS4	ND	ND	**2.643**	7.66E‐12
ABCA7	0.693	3.20E‐07	0.699	3.12E‐05
CR1	ND	ND	0.133	NS
TSPAN14	−0.030	NS	0.146	2.07E‐02
CTSH	0.269	1.09E‐04	−**1.788**	7.73E‐15
MYO15A	**2.571**	1.60E‐09	ND	ND
SIGLEC11	ND	ND	ND	ND
ADAM17	0.395	2.54E‐11	0.408	7.43E‐10
WDR81	0.123	1.06E‐03	0.273	3.89E‐04
RBCK1	**1.169**	1.22E‐20	−0.108	NS
ICA1	−0.975	2.35E‐16	**1.206**	1.27E‐09
CD2AP	−0.543	3.84E‐10	0.105	NS
SLC2A4RG	0.515	5.14E‐12	−**1.034**	2.01E‐14
EED	−0.874	1.22E‐16	−0.147	6.80E‐03
RHOH	ND	ND	ND	ND
MAF	ND	ND	0.621	NS
PTK2B	**1.098**	3.25E‐16	−0.030	NS
DOC2A	−0.786	3.47E‐07	0.312	8.42E‐03
SPI1	ND	ND	ND	ND
ACE	−0.124	3.25E‐03	−0.675	1.28E‐02
TNIP1	−0.077	2.31E‐02	0.290	3.70E‐05
CD33	ND	ND	ND	ND
ANK3	0.045	NS	0.398	1.04E‐03
BIN1	−0.227	6.30E‐07	−**1.73**	2.10E‐14
ABI3	ND	ND	0.521	1.34E‐02
JAZF1	**1.176**	5.06E‐18	0.109	NS
EPHA1	ND	ND	0.642	1.41E‐03
GRN	0.951	6.52E‐17	0.920	1.67E‐12
CLU	**1.714**	6.42E‐24	**1.300**	1.22E‐09
PLCG2	ND	ND	**1.108**	3.25E‐09
IDUA	**2.427**	3.74E‐16	0.424	3.71E‐02
PLEKHA1	−0.092	4.58E‐2	0.587952	1.01E‐09
TMEM106B	**1.379**	2.96E‐19	0.621	1.99E‐06
PLD3	0.310	2.31E‐8	−0.094	NS
PICALM	−0.029	NS	−0.128	NS

The genes listed from CTSB and below are from Andrews et al.^18^ Genes with a log_2_ FC ≥ 1 are bolded. NS, not significant; ND, not detected.

**FIGURE 2:**
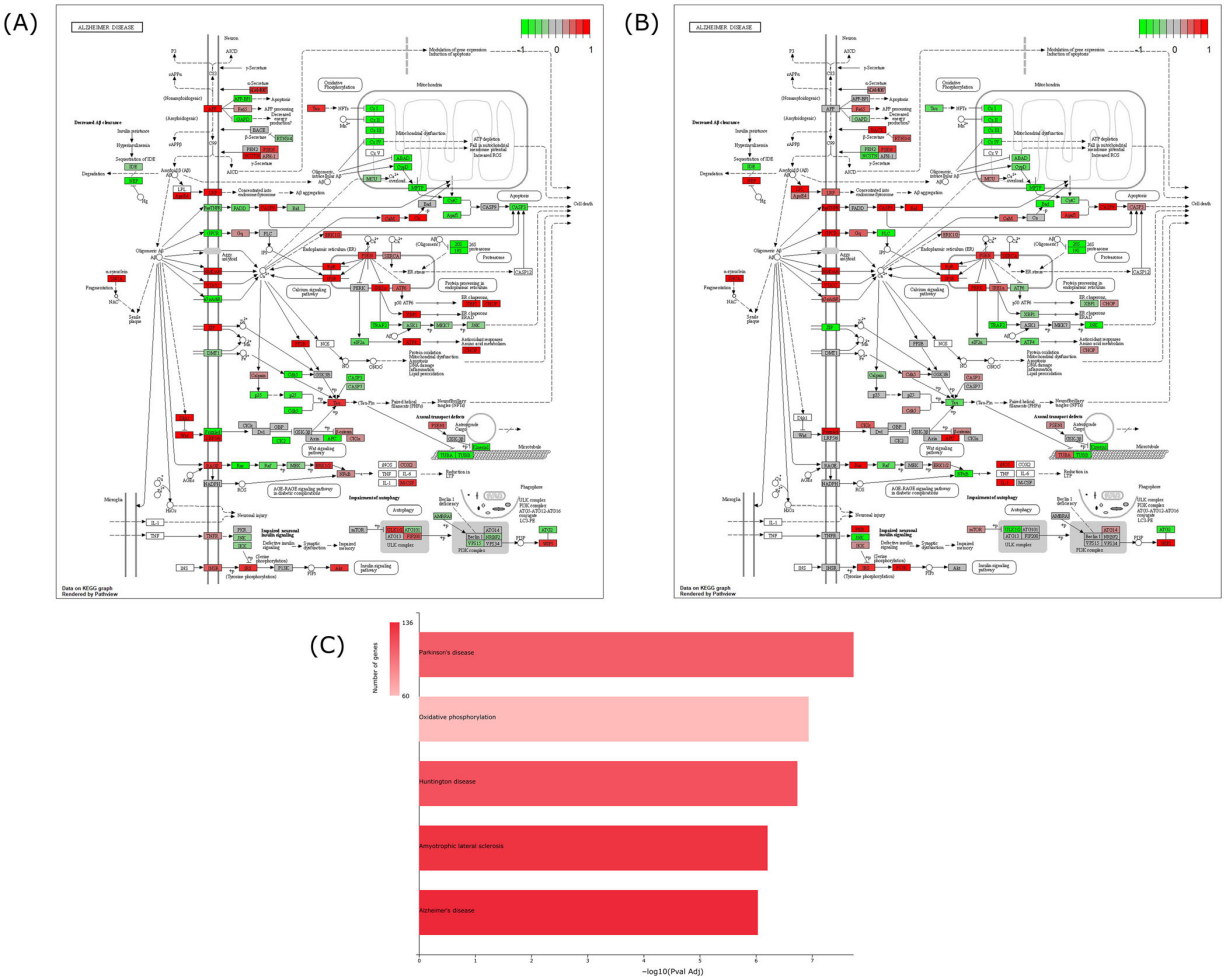
The AD pathway in Kyoto Encyclopedia of Genes and Genomes (KEGG) and the most enriched KEGG pathways defined by the Equivalent Change Index (ECI) gene set. (A) The KEGG AD pathway for the SH‐SY5Y ρ0 versus ρ+ cell RNA‐seq. (B) The KEGG AD pathway for the NT2 ρ0 versus ρ+ cell RNA‐seq. (C) Top five (most statistically significant) KEGG pathways from the SH‐SY5Y and NT2 expression ECI analysis.

As the KEGG database features disease‐specific pathways, we performed a GSEA using that database to specifically consider the enrichment of genes deemed pertinent to AD biology. SH‐SY5Y ρ0 and NT2 ρ0 altered genes were significantly enriched in the KEGG database *Alzheimer disease* pathway (FDR = 1.79E‐02 for the SH‐SY5Y ρ0 cells; FDR = 9.41E‐03 for the NT2 ρ0 cells), which indicates in human neuronal cell lines, mtDNA depletion alters AD biology, at least as it is defined in that database. Figure 2A,B shows the resulting pattern of KEGG *Alzheimer's disease* pathway upregulated and downregulated genes.

We also looked at KEGG pathways significantly enriched with genes that had a high ECI value in the SH‐SY5Y and NT2 ρ0 lines. Four of the five most enriched pathways from this analysis were neurodegenerative disorders (*Alzheimer's disease*, *amyotrophic lateral sclerosis*, *Parkinson's disease*, and *Huntington's disease*), which link primary mitochondrial dysfunction to a spectrum of neurodegenerative diseases (Figure 2C). The *Alzheimer's disease* pathway included 136 of the 5000 DEGs with the highest ECI scores.

### 3.3 Reactome database GSEA and ORA identify AD‐pertinent pathway changes

**FIGURE 3:**
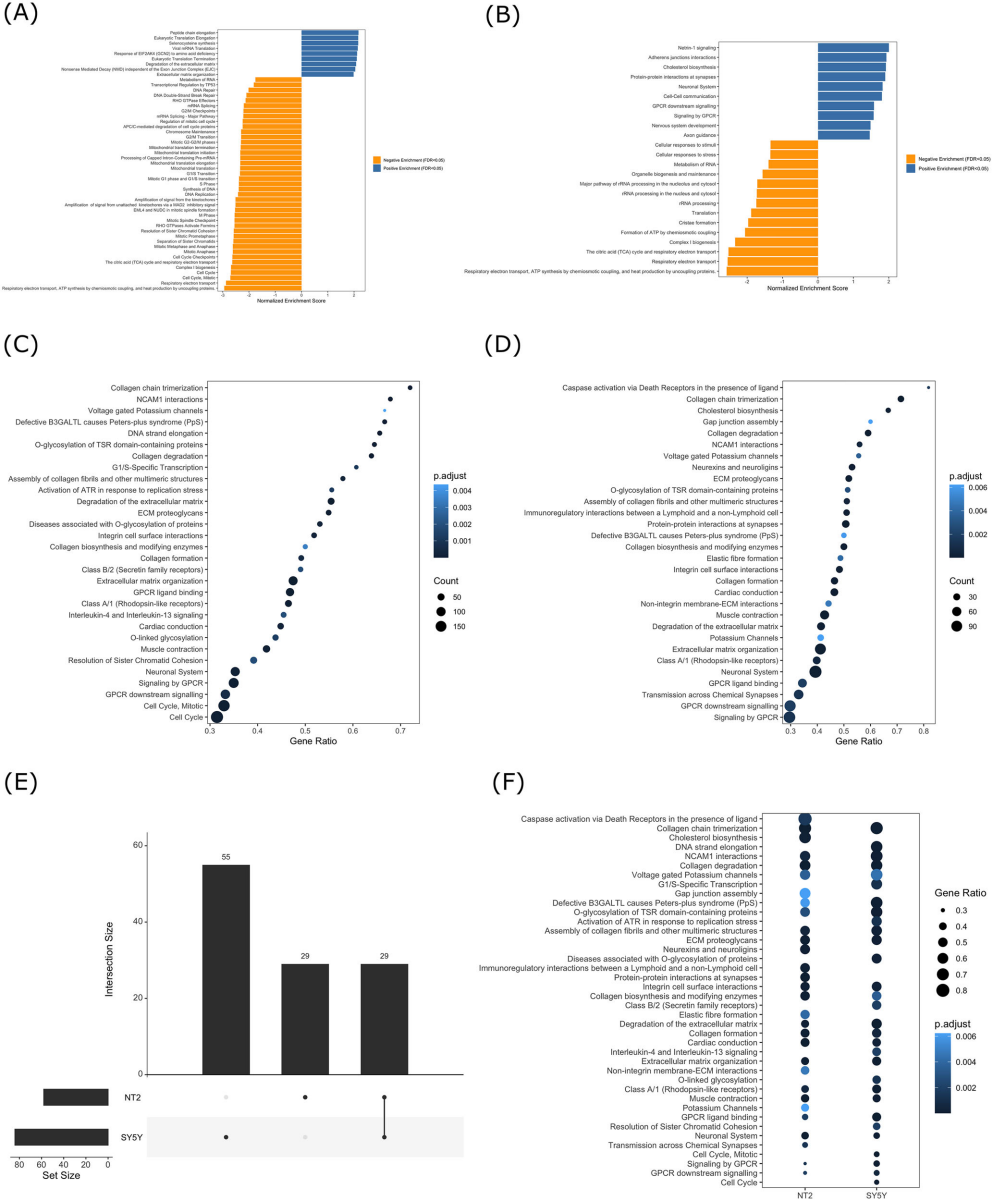
Gene set enrichment analysis (GSEA) and overrepresentation analysis (ORA) using Reactome. (A) SH‐SY5Y GSEA analysis, 30 most significantly enriched pathways. (B) NT2 GSEA analysis, all 24 significantly enriched pathways. (C) SH‐SY5Y ORA analysis, 30 most significantly enriched pathways. (D) NT2 ORA analysis, 30 most significantly enriched pathways. (E) Proportion of overlapping ORA pathways. (F) SH‐SY5Y and NT2 ORA overlap.

GSEA and ORA analyses, performed using the Reactome database, showed the enrichment of pathways that directly reflect the state of the mitochondrial respiratory chain, the functional parameter our experimental manipulation targeted (Figure 3). The impact on mitochondria, however, was not limited to the respiratory chain and included, for example, several pathways specifically related to SH‐SY5Y cell mitochondrial translation (Figure 3A). Beyond this, the Reactome GSEA and ORA additionally identified multiple pathways whose defining genes are not considered mitochondria‐related genes.

For the SH‐SY5Y ρ0 to ρ+ cell comparison, the GSEA identified 256 significant pathways (Table 1). Table S4 lists all significant SH‐SY5Y GSEA Reactome pathways, and Figure 3A shows the 50 most enriched pathways. The SH‐SY5Y pathways most enriched with upregulated genes (normalized enrichment scores (NES) exceeding 2) were highly pertinent to proteostasis and included *peptide chain elongation*, *eukaryotic translation elongation*, *eukaryotic translation termination*, *response of EIF2AK4 (GCN2) to amino acid deficiency*, and *viral mRNA translation*. The context for the *viral mRNA translation* pathway comes from a recent proteomics study that reported prominent enrichment of viral‐related pathways in APOE ε4 carriers with and without clinical AD.^25^ To add context to the *response of EIF2AK4 (GCN2) to the amino acid deficiency* pathway, a key mediator of EIF2AK4 activity is the activating transcription factor 4 (ATF4)^26^; the SH‐SY5Y ρ0 cell ATF4 log_2_FC was 1.06 (FDR = 4.98E‐18).

Multiple SH‐SY5Y GSEA pathways that were defined by downregulated DEGs related to RNA biology. Specific pathways include *metabolism of RNA*, *processing of capped intron‐containing pre‐mRNA*, and *mRNA splicing*.

The NT2 ρ0 versus ρ+ cell GSEA revealed 24 significantly enriched pathways (Figure 3B and Table S4). The *cholesterol biosynthesis* pathway was enriched with genes upregulated in ρ0 cells. Multiple altered pathways pertained to neuronal signaling and development. The *Netrin‐1 signaling*, *neuronal systems*, *axon guidance*, and *nervous system development* pathways were each enriched with NT2 ρ0 cell‐upregulated genes. The netrin‐1 gene (NTN1), a member of the *Netrin‐1 signaling* pathway and a key regulator of axonal guidance during nervous system development,^27^ was increased in the NT2 ρ0 cells (log_2_FC = 1.36; FDR = 5.03E10‐13), but this gene was not detected in SH‐SY5Y cells. Eleven GSEA pathways were concordantly altered in the NT2 and SH‐SY5Y ρ0 cells.

The SH‐SY5Y cell ORA identified 84 significant pathways. Table S4 lists all significant pathways, and Figure 3C shows the 30 most enriched pathways. Multiple pathways in Figure 3C associate with chemical signaling, such as the *signaling by GPCR*, *GPCR ligand binding*, *GPCR downstream signaling*, and *class B/2 (secretin family receptors)* pathways. This pattern of change suggests a robust impact on neurotransmission and receptor‐mediated responses. In general, changes to signaling pathways imply mitochondrial function is relevant to those pathways in our models, but the direct or indirect nature of that relevance or its extrapolation to human brains is to some extent unclear.

The NT2 ORA revealed 58 significantly enriched pathways. Table S4 lists all significant pathways, and Figure 3D shows the 30 most enriched pathways. Several of the pathways in Figure 3D relate to synaptic structure and function, such as the *neurexins and neuroligins*, *protein–protein interactions at synapses*, *transmission across chemical synapses*, and *potassium channels* pathways. Central to these findings are the pathways involving neurexins (NRXNs) and neuroligins (NLGNs), which are crucial synaptic cell‐adhesion molecules. These proteins are integral to both excitatory glutamatergic and inhibitory GABAergic synapses in the mammalian brain.^28^

A comparative analysis of the SH‐SY5Y ρ0 and NT2 ρ0 cell ORA‐identified Reactome pathways showed 55 pathways were altered in just the SH‐SY5Y ρ0 cells, 29 were altered in just the NT2 ρ0 cells, and 29 pathways were altered in both (Figure 3E). Figure 3F shows the results of this comparative analysis over 40 pathways. The *neuronal system* pathway, a broad network encompassing 419 genes involved in synaptic signaling and neural activity, was significantly enriched in both lines. Both lines showed alterations in several G‐protein coupled receptor (GPCR)‐associated pathways and neural cell adhesion molecule 1 (NCAM1)‐mediated cell adhesion (the *NCAM1 interactions* pathway), further implicating disrupted communication at neuronal interfaces.^29^, ^30^

### 3.4 GO database GSEA and ORA identify AD‐pertinent term changes

**FIGURE 4:**
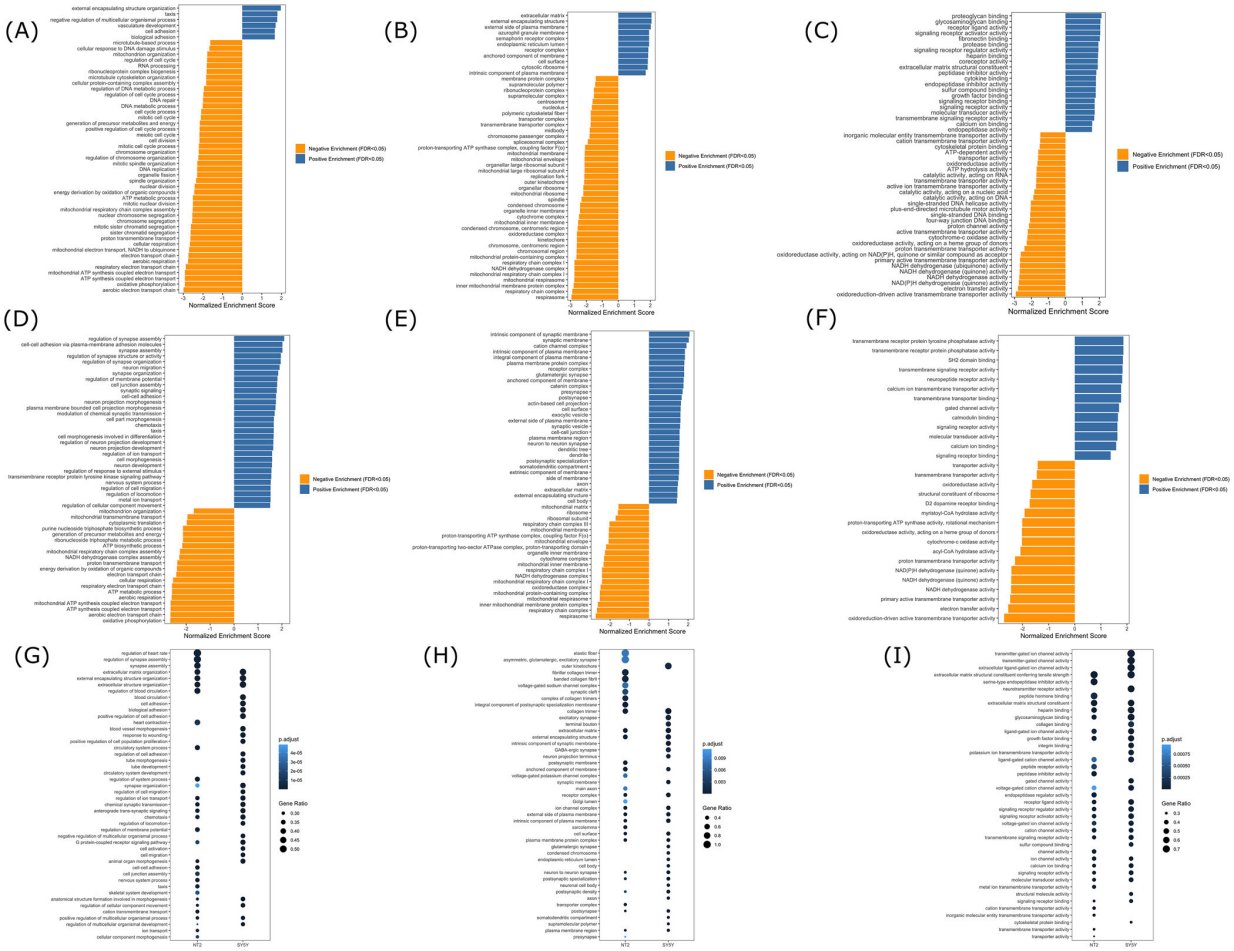
Gene set enrichment analysis (GSEA) and overrepresentation analysis (ORA) using Gene Ontology (GO). For (A) through (F), blue bars indicate enrichment due to upregulated genes, and gold bars indicate enrichment due to downregulated genes. (A) Biological process SH‐SY5Y GSEA analysis, 50 most significantly enriched terms. (B) Cell component SH‐SY5Y GSEA, 50 most significantly enriched 50 terms. (C) Molecular function SH‐SY5Y GSEA analysis, 50 most significantly enriched terms. (D) Biological process NT2 GSEA analysis, 50 most significantly enriched terms. (E) Cell component NT2 GSEA analysis, 50 most significantly enriched terms. (F) Molecular function NT2 GSEA analysis, 50 most significantly enriched terms. (G) Thirty most significantly enriched terms from biological process SH‐SY5Y and NT2 ORA analyses. (H) Thirty most significantly enriched terms from cell components SH‐SY5Y and NT2 ORA analyses. (I) Thirty most significantly enriched terms from molecular functions SH‐SY5Y and NT2 ORA analyses.

GSEA and ORA revealed multiple altered GO terms (Table 1 and Figure 4). For the SH‐SY5Y ρ0 cells, GSEA identified 503 significant terms, including 330 from the Biological Process category, 96 from the Cell Component category, and 77 from the Molecular Function category. For the NT2 ρ0 cells, GSEA identified 173 significant terms, including 88 from the Biological Process category, 55 from the Cell Component category, and 30 from the Molecular Function category. Table S5 lists all significant terms. Reminiscent of the Reactome analyses, in addition to the enrichment of respiratory chain activity‐related terms, we saw an enrichment of non‐respiratory chain mitochondrial terms that feature nuclear encoded genes, such as the *mitochondrial envelope* term (Figure 4B). The GO GSEA and ORA also identified many terms whose defining genes are not considered mitochondria‐related genes.

For the SH‐SY5Y ρ0 to ρ+ comparison, Figure 4A–C shows the 50 most enriched GSEA terms from each of the three GO categories. In each case, there were more terms predominantly enriched with downregulated DEGs. Biological Process terms enriched with downregulated ρ0 genes included *RNA processing* and *ribonucleoproteins complex biogenesis*, which reflects a theme seen in the Reactome analysis. Microtubule‐based processes and cytoskeletal organization terms were predominantly enriched with downregulated genes, despite the fact that the SH‐SY5Y ρ0 cells showed a 1.7‐fold increase in microtubule‐associated protein tau (MAPT) expression (log_2_ FC = 0.77; FDR = 5.26E‐14). In the Cellular Component category, the *cytosolic ribosome* and *endoplasmic reticulum lumen* terms both exhibited enrichment of predominantly upregulated genes, which is consistent with the translation biology modifications seen in the Reactome GSEA. The *Semaphorin receptor complex* term, which is relevant to axon guidance,^31^ was also enriched with upregulated genes. The Molecular Function category showed an upregulated gene enrichment of the *heparin binding* term, which is relevant to Aβ aggregation,^32^ as well as the immune response‐relevant *cytokine binding* term.^33^ Other Molecular Function terms included the *ATP‐dependent activity* and *ATP hydrolysis activity* terms, which likely reflect a response to energy stress, and could affect a variety of cell processes, including endosome biology.^34^

Figure 4D–F shows the 50 most enriched NT2 ρ0 cell GSEA terms from the Biological Process and Cell Component categories and the 30 most enriched NT2 ρ0 terms from the Molecular Function category. For the Biological Process and Cell Component categories, the NT2 ρ0 cells featured a higher proportion of terms enriched with upregulated genes than the SH‐SY5Y ρ0 cells. The Biological Process category included the *regulation of the cellular component movement* term, which is highly pertinent to endosome activity.^35^ The Cellular Component category showed an upregulated gene enrichment of the *catenin complex* term, which has ties to Wnt signaling^36^ and the *exocytic vesicle* term, which is pertinent to the movement of proteins and lipids within a cell.^37^ The Molecular Function category showed an upregulated gene enrichment of the *calmodulin binding domain* term, which influences phosphatase and kinase activity,^38^ and a downregulated enrichment of the *acyl CoA hydrolase activity* term, which plays a role in lipid biology.^39^ It also showed an upregulated gene enrichment of the *transmembrane receptor binding tyrosine phosphatase activity* term that modulates protein phosphorylation within cells and is functionally relevant to insulin signaling.^40^

Of the 503 GO‐based GSEA SH‐SY5Y terms and 173 NT2 terms, 88 were shared terms. This included 39 Biological Process, 32 Cell Component, and 17 Molecular Function terms.

The ORA‐based GO analysis showed themes that reflect those seen in the GSEA (Figure 4G–I). The Biological Process category identified synapse and GPCR signaling terms for both SH‐SY5Y ρ0 and NT2 ρ0 cells, and the Cellular Component category also yielded synapse‐related terms. In the Molecular Function category, both cell lines showed significant enrichment of the *heparin‐binding* term. Enrichment of the *receptor ligand activity* term in the Molecular Function category was present for both ρ0 cell types, both cell types showed enrichment of the Cell Component *intrinsic component of plasma membrane* term, and the NT2 ρ0 cells showed enrichment of the Cell Component *transporter complex* term. These three terms are pertinent to endosome biology.^41^

### 3.5 IPA database ORA and *z*‐scores identify AD‐pertinent pathway changes

**FIGURE 5:**
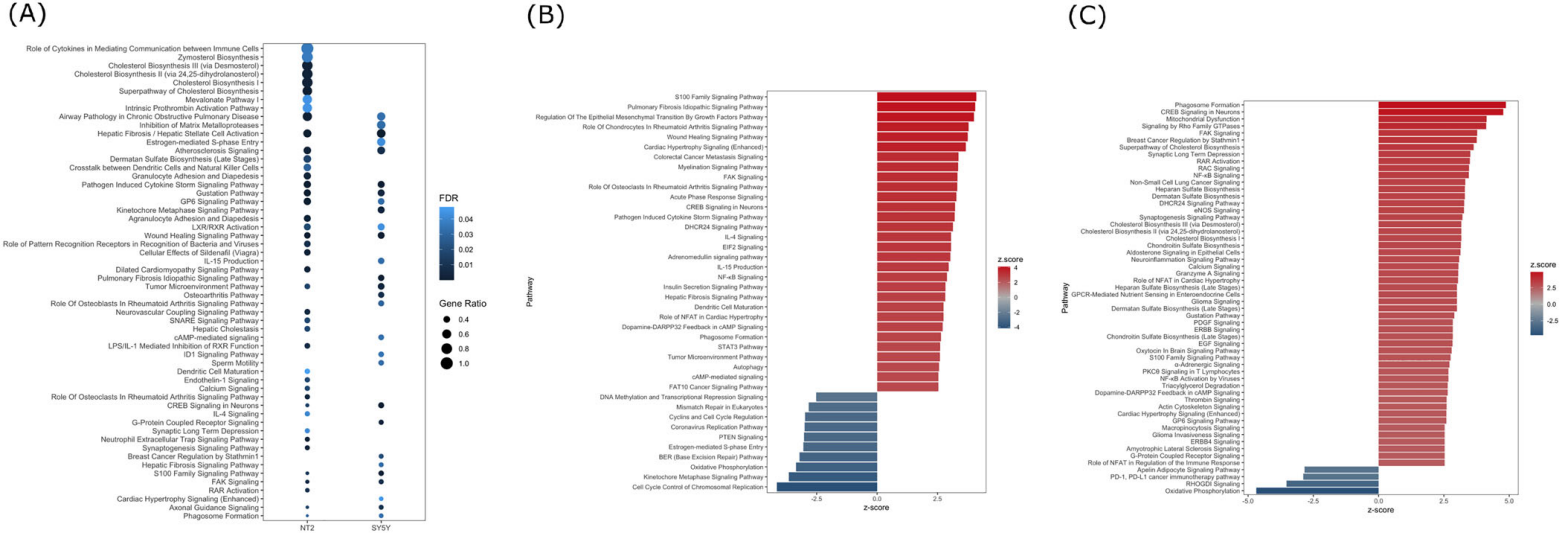
Overrepresentation analysis (ORA) and z‐score analyses using Ingenuity Pathway Analysis. (A) Comparative ORA results for 56 pathways. (B) For SH‐SY5Y cells, the 40 most significantly enriched *z*‐scores from the 78 pathways with an absolute activation *z*‐score of >2 . (C) For the NT2 cells, the 56 most significantly enriched *z*‐scores from the 102 pathways with an absolute activation *z*‐score of > 2.

An ORA of the IPA database revealed 14 pathways that were uniquely altered in the SH‐SY5Y ρ0 cells, 28 pathways that were uniquely altered in the NT2 ρ0 cells, and 14 pathways that were altered in both (Figure 5A and Table S6). Affected pathways associated with the immune system and inflammation, synapse biology, lipid biology, endosome biology, and insulin biology. One of the lipid biology‐related pathways in both cell types included the *LXR/RXR activation pathway*.^42^ Both models showed enrichment of the *axonal guidance signaling* pathway that is pertinent to synapse biology and of the *S100 family signaling pathway* that is relevant to inflammation.^43^ The SH‐SY5Y ρ0 cells revealed enrichment of the oxidative stress^44^ and innate and adaptive immune system‐relevant *cellular effects of sildenafil (Viagra)* pathway.^45^ The NT2 ρ0 cells showed enrichment of the *crosstalk between dendritic cells and natural killer cells* pathway, which also impacts both innate and adaptive immunity,^46^ and of the *SNARE signaling pathway*, which impacts endosome function.^47^

The IPA revealed 78 pathways with an absolute *z*‐score ≥ 2 in the SH‐SY5Y ρ0 versus ρ+ cells and 102 pathways in the NT2 ρ0 versus ρ+ cells (Figure 5B,C). The SH‐SY5Y ρ0 cells showed activation of several inflammation/immune system‐related pathways, such as the *IL‐4 signaling*, *IL‐15 production*, and *NF‐κB signaling* pathways. They also showed activation of the *autophagy* pathway and the *EIF2 signaling* pathway, the latter of which mediates the integrated stress response.^48^ The activation of these two pathways suggests a compensatory response to protein stress. The *cAMP‐mediated signaling* pathway, which is pertinent to cAMP response element binging protein (CREB) function,^49^ and the *CREB signaling in neurons* pathway itself were activated. The SH‐SY5Y *z*‐score analysis also showed modifications to insulin biology‐related pathways; there was activation of the *insulin secretion signaling pathway* and inhibition of the *PTEN signaling pathway*.^50^

The NT2 ρ0 cells showed activation of the *neuroinflammation signaling pathway* and other inflammation/immune system‐related pathways and of the insulin biology‐relevant *signaling by the rho family GTPases* pathway.^51^ The *calcium signaling* pathway and the related *CREB signaling in neurons* pathway, which is influenced by or influences energy metabolism, synapse, lipid, insulin signaling, and inflammation/immune system biology,^49^ were robustly activated in the NT2 ρ0 cells.

## 4 DISCUSSION

Human neuronal cells experiencing primary mitochondrial dysfunction due to chronic mtDNA depletion show pervasive transcriptional changes and a reshuffling of nuclear gene expression. This includes changes to genes implicated in AD, or whose protein products are altered in AD.^18^, ^19^, ^20^ The impact on the expression of AD‐relevant genes is extensive, and the transcriptome changes touch multiple AD molecular and physiologic phenomena.^11^ We consequently conclude primary mitochondrial dysfunction, or at least human neuronal cell line mitochondrial dysfunction caused by chronic mtDNA depletion, alters gene expression in ways that recapitulate or predictably promote AD phenomena.

Gene expression studies frequently elevate the importance of larger changes, especially those that more than double or halve expression levels. Less than 25% of the genes in both lines met this threshold. While this is far less than the ∼80% of genes with some degree of altered expression, we caution against dismissing the importance of the smaller changes. In an insidious disease such as AD that evolves over decades,^52^ subtle changes, singly or in combination, could play a role. Accordingly, we interrogated our RNA‐seq data from both GSEA and ORA perspectives.

Our ρ0 lines increased the expression of genes that encode proteins that aggregate in AD, including tau and α‐synuclein.^23^, ^53^ Multiple factors determine whether a protein aggregates, and protein abundance is one of them.^54^ This raises the possibility that a mitochondria dysfunction‐induced change in the transcription of certain genes contributes to AD protein aggregation. The expression of genes linked to autosomal dominant AD, such as APP and PSEN1,^18^ also changed. PSEN1 processes APP potentially toward Aβ,^55^ which could contribute to Aβ aggregation. Our data further revealed a response to protein stress, which implies that in our models, mitochondrial dysfunction induces protein stress.

AD‐focused omics studies report robust enrichment of mitochondria‐related pathways.^9^, ^10^, ^11^, ^12^ Our ρ0 cells showed profound reductions in pathways pertinent to oxidative phosphorylation and the respiratory chain, which was expected as mtDNA depletion, our experimental manipulation, precludes expression of mtDNA‐encoded respiratory chain subunits. However, nuclear DNA‐encoded mitochondrial genes also downregulated their expression. Mitochondria‐nucleus communication channels must exist to coordinate this. Mitochondrial pathways downregulated in our study recapitulate downregulated pathways seen in omics studies of AD brain.^11^

Our findings align with reports of altered AD brain synaptic proteins.^11^, ^12^, ^56^, ^57^ Both ρ0 lines showed changes in GPCR signaling and neurexin/neuroligin‐mediated synaptic adhesion that relate to known changes in AD synaptic function.^58^, ^59^, ^60^ The netrin‐1 signaling pathway, which influences axon guidance and synaptogenesis, was enriched with upregulated genes, which is consistent with a report of this pathway and the NTN1 gene in AD brains.^27^ NTN1 also interacts with secretases that process APP, which could link this activation to AD histopathology.^59^

Our study showed enrichment of inflammation‐ and immune response‐relevant gene expression and, by extension, inflammation‐ and immune response‐affiliated pathways. This is not surprising as mitochondria influence inflammation and immune function through multiple mechanisms.^61^ Brain‐based studies commonly emphasize changes to microglia,^20^ which constitute the brain's innate immune system and produce inflammatory cytokines.^62^ Our neuronal cell lines lack characteristics typical of microglia, such as the expression of the AD‐associated TREM2 gene, which makes our observed inflammation and immune system parameters even more notable.

Polymorphisms in APOE influence AD risk.^63^ Several hypotheses attempt to explain this in ways that extend beyond its role in brain lipid homeostasis.^21^ Under normal circumstances, neurons produce very little APOE.^64^ We previously reported that SH‐SY5Y ρ0 cells dramatically increased APOE expression,^7^ which this study confirmed. AD GWAS also ties genes that affect lipid handling and homeostasis to AD.^18^, ^19^, ^20^ In general, it is difficult to functionally separate lipid and mitochondrial biology, as mitochondria launder carbon that promotes lipid synthesis, and phospholipid synthesis occurs at mitochondrial‐associated membranes that form between mitochondria and endoplasmic reticulum.^65^ Our study indicates that primary mitochondrial dysfunction alters neuronal cell lipid biology.

Published studies highlight perturbed RNA dynamics in AD.^66^, ^67^ Both ρ0 lines showed direct and profound changes to RNA biology. The SH‐SY5Y ρ0 cells showed a specific pattern of preferentially upregulated ribosomal genes but downregulated RNA‐processing pathways.

GWASs implicate a role for endosome function and identified an association between AD and the BIN1 gene.^18^, ^20^ Changes in levels of genes and proteins that mediate insulin signaling, in conjunction with epidemiologic studies, implicate relationships between AD and type 2 diabetes.^68^ Our pathway analyses indicated that primary mitochondrial dysfunction influenced endosome biology and insulin signaling.

Primary mitochondrial dysfunction impacted signal transduction pathways. Some, like the CREB signaling pathway, affect many of the AD‐associated biological phenomena we focused on.^69^ CREB is a transcription factor that, following serine 133 phosphorylation, binds DNA to modify gene expression.^49^ Serine phosphorylation is mediated by protein kinase A (PKA), which is activated through various events that include GPCR signaling (a pathway enriched in our study) but especially cyclic adenosine monophosphate (cAMP). cAMP is generated by adenylyl cyclase enzymes whose activities are differentially affected by cytosolic calcium,^49^ and mitochondria influence cytosolic calcium levels.^70^ Although the IPA also revealed enrichment of specific cAMP and calcium signaling‐related pathways, our study cannot establish whether this series of events indeed occurred.

While our analyses of two neuronal ρ0 human lines implicated similar themes, changes were frequently documented in one line but not the other, and some changes moved in different directions. Divergence likely reflects inherent differences between the SH‐SY5Y and NT2 lines. Investigators tend to differentiate SH‐SY5Y cells into cholinergic and dopaminergic neurons and NT2 cells into glutamatergic and GABAergic neurons. The SH‐SY5Y line originated from a female and the NT2 line from a male. Omics studies indicate AD molecular signatures are sex and neuron subtype‐dependent.^13^, ^71^, ^72^

Using two cell lines allowed us to identify the equivalently changed genes and to define pathways that these genes enriched. In addition to identifying AD as an enriched pathway, there was enrichment of the *Parkinson's disease*, *amyotrophic lateral sclerosis*, and *Huntington's disease* pathways. Like AD, those diseases feature mitochondrial dysfunction.^73^ Our data support the view that mitochondrial dysfunction could functionally link these neurodegenerative diseases.

Although findings across our different analytical approaches tend to reinforce each other, we observed features specific to one analytic approach but not another. Changes in dividing ρ0 cells also may not extrapolate well to AD brains. Experiments exploring other types of mitochondrial dysfunction in other models, for example, mtDNA transcription inhibition in induced pluripotent stem cell‐derived neurons, could help distinguish AD‐relevant from non‐relevant changes.

Our study induced primary mitochondrial dysfunction through chronic mtDNA depletion. The stability of the ρ0 cell defect ensures time to adapt. For a chronic and slowly progressing disorder such as AD, there are advantages to using chronic over acute manipulations. Also, overall mtDNA levels in AD brains are lower than in controls.^74^ We recently showed, in brains studied by the Accelerated Medicines Partnership in AD (AMP‐AD) consortium, an inverse relationship between mtDNAcn and AD neuropathologic change (the extent of plaques and tangles). In that study, the brains with the highest mtDNAcn had the least AD neuropathologic change. Conversely, a direct relationship existed between mtDNAcn and cognitive test performance, as brains from donors that performed better on cognitive testing prior to death contained more mtDNAcn than brains from donors that performed at a lower level.^74^

Our models have limitations. A major disadvantage is their tumor derivation. Unlike neurons, SH‐SY5Y and NT2 ρ0 and ρ+ cells grow and divide. It is difficult to know how well changes observed in cell growth, division, and cycling extrapolate to AD or if those changes represent unrelated modifications to tumor‐specific programs. Additionally, neuronal tumor lines exhibit bioenergetic characteristics that differ from genuine neurons, such as high rates of aerobic glycolysis.^75^

The magnitude of ρ0 cell mitochondrial defects, for example reductions in respiratory chain ATP production, should exceed defects in surviving neurons from AD brains. Non‐mitochondrial changes in our system also presumably reflect finely tuned adaptive responses. While most biological changes in AD may similarly represent adaptive responses or attempts to adapt, ρ0 cells predictably succeed more often than AD brains. Succeeding and failing systems could manifest opposing shifts. It is probably reasonable, therefore, to focus on the identification of shared pathways and DEGs, as opposed to the magnitude and direction of change.

The extent to which our ρ0 cell lines model AD depends on the extent to which primary mitochondrial dysfunction drives AD. This question remains unsettled. We feel it is best to consider our ρ0 cell lines not as models of AD but rather models of primary mitochondrial dysfunction.

Despite these limitations, our study shows that primary mitochondrial dysfunction impacts multiple AD‐associated biological events. It links an age‐associated biological phenomenon, declining mitochondrial function,^3^ to an AD‐specific, gene expression‐defined molecular landscape. Our study is consistent with a mitochondrial cascade hypothesis that proposes mitochondrial dysfunction initiates AD molecular changes.^1^ Much of this is not surprising given prior research, but our approach provides a unique perspective. This study also provides a novel dataset that can support hypothesis generation.

## CONFLICT OF INTEREST STATEMENT

The authors have no conflicts of interest to report. All author disclosures are available in the Supporting Information.

## CONSENT STATEMENT

No consent was necessary.

## Supporting information



Supporting Information

Supporting Information

Supporting Information
