# Supplementary material for: MtDNA‐depleted neuronal cell transcriptomes reveal Alzheimer's disease‐related changes
Source: Alzheimers Dement. 2025 Nov 26;21(11):e70929. doi: 10.1002/alz.70929 (PMC12647984; doi:10.1002/alz.70929)
Supplement: Supplementary file 1 — Supporting Information [file ALZ-21-e70929-s003.docx]

**Supplemental Material**

| **Supplementary Table 1. Relative expression of the 13 mtDNA-encoded structural genes.** | | | | |
| --- | --- | --- | --- | --- |
| **Gene** | **SH-SY5Y ρ0 Cells** | | **NT2 ρ0 Cells** | |
|  | **Log_2_FC** | **FDR** | **Log_2_FC** | **FDR** |
| MT-ND1 | -17.606 | 5.17E-34 | -14.704 | 1.77E-23 |
| MT-ND2 | -16.585 | 3.70E-30 | -14.784 | 5.15E-24 |
| MT-ND3 | -16.247 | 2.43E-32 | -15.002 | 1.20E-26 |
| MT-ND4 | -16.089 | 9.58E-34 | -16.089 | 3.80E-26 |
| MT-ND4L | -15.948 | 4.29E-35 | -14.258 | 6.66E-27 |
| MT-ND5 | -15.426 | 4.81E-32 | -13.788 | 9.54E-26 |
| MT-ND6 | -14.159 | 1.98E-32 | -13.169 | 3.54E-25 |
| MT-CO1 | -16.806 | 2.41E-29 | -14.949 | 7.36E-22 |
| MT-CO2 | -16.898 | 5.62E-33 | -14.453 | 9.90E-26 |
| MT-CO3 | -16.730 | 6.16E-32 | -14.656 | 9.90E-26 |
| MT-CYB | -16.305 | 2.02E-33 | -14.178 | 1.39E-25 |
| MT-ATP6 | -16.648 | 5.20E-32 | -15.146 | 5.94E-25 |
| MT-ATP8 | -16.433 | 4.53E-34 | -14.140 | 9.90E-26 |

**Supplementary Tables 2-6:** See excel spreadsheet.
